# Quality Evaluation of Large Language Model–Assisted Generation of Initial Senior Physician Ward Round Records for Patients With Acute Poisoning: Cross-Sectional Study

**DOI:** 10.2196/91222

**Published:** 2026-06-30

**Authors:** Junping Zhu, Wei Pan, Yonghong Wang, Kui Yan, Zhicheng Fang, Xianyi Yang

**Affiliations:** 1 Department of Emergency Medicine, Taihe Hospital, Hubei University of Medicine, Renmin South Road 32, Maojian District, Shiyan, Hubei, 442012, China, 86 13593776564; 2 Hubei Provincial Clinical Medical Research Center for Pneumoconiosis and Poisoning, Hubei Provincial Hospital of Integrated Chinese & Western Medicine, Wuhan, Hubei, China

**Keywords:** large language models, generative artificial intelligence, emergency department, ward round records, clinical documentation, acute poisoning, medical records, DeepSeek, ChatGPT, artificial intelligence, AI

## Abstract

**Background:**

Large language models (LLMs) have shown potential in medical text generation. Senior physician ward round records are critical documents whose quality reflects the accuracy and continuity of clinical decision-making. The initial record is particularly important, as it represents the first formal senior-level synthesis of a patient’s presentation, establishing the diagnostic framework and treatment direction for all subsequent care. The quality of LLM-generated initial records for acute poisoning remains unclear.

**Objective:**

Focusing on patients with acute poisoning, this study systematically compared medical record writing quality among DeepSeek, ChatGPT (OpenAI), and human physicians to clarify the clinical value of LLMs.

**Methods:**

A retrospective analysis included 256 cases of acute poisoning from the emergency department ward of Taihe Hospital, Hubei University of Medicine. DeepSeek-V3.2-Exp and GPT-5.1 generated senior physician ward round records from standardized Chinese-language prompts, which were compared with the original medical charts. Blinded evaluations were performed by 3 senior emergency physicians, who scored overall quality across 5 dimensions on a Likert scale (from 1 to 5): case characteristics, current diagnosis, differential diagnosis, treatment plan, and prognosis assessment. Error frequencies were documented under 3 categories (inaccuracies, omissions, and fabrications), and potential harm was assessed using a modified Agency for Healthcare Research and Quality harm scale.

**Results:**

DeepSeek achieved the highest mean total score (24.14, SD 0.90), which was significantly higher than ChatGPT (23.30, SD 1.42; *P*<.001) and the physician group (23.86, SD 0.86; *P*=.02). DeepSeek had the highest score for differential diagnosis (mean 4.98, SD 0.10) and prognosis assessment (mean 4.73, SD 0.42) and was comparable to physicians in case characteristics (DeepSeek: mean 4.90, SD 0.23; physicians: mean 4.96, SD 0.15; *P*>.001). For drug and pesticide poisoning, DeepSeek's mean total scores (24.23, SD 0.75 and 23.92, SD 1.14, respectively) were significantly higher than ChatGPT’s (23.34, SD 1.33 and 22.78, SD 1.33, respectively; *P*<.001 for both). In biological toxin poisoning, DeepSeek (mean 23.97, SD 0.96) and physicians (mean 24.26, SD 0.62) scored similarly, both significantly higher than ChatGPT (mean 22.53, SD 1.86; *P*<.001). Overall potential harm scores were low across all 3 groups (<1 point), without significant differences (*P*=.38), although high-harm records were significantly more frequent in both LLM groups than in the physician group (*P*=.02).

**Conclusions:**

LLMs performed satisfactorily in generating initial senior physician ward round records for acute poisoning, with DeepSeek particularly outperforming the physician group in differential diagnosis and prognosis assessment and showing potential to assist clinical documentation. However, the significantly higher proportion of high-harm errors in LLM-generated records underscores the need for mandatory physician review before incorporation into official medical records.

## Introduction

Acute poisoning is a common critical condition in emergency departments, characterized by rapid progression and high mortality rates, which places significant demands on clinicians’ rapid diagnostic and management capabilities [1]. Senior physician ward rounds serve as a core component of medical quality control, and senior physician ward round records are essential medical documents for organizing clinical information and clarifying diagnostic and therapeutic directions. They also serve as critical evidence for teaching, quality traceability, and medical dispute resolution [2]. Among these documents, the initial senior physician ward round record is of particular significance: it represents the first formal synthesis of a patient’s clinical presentation by a senior physician, establishing the diagnostic framework and treatment direction that guides all subsequent care. Its quality is, therefore, directly linked to patient safety and care continuity. However, heavy clinical workloads often prevent physicians from ensuring the standardization and completeness of ward round records, thereby affecting the quality of care [3].

In recent years, large language models (LLMs) such as ChatGPT and DeepSeek have been increasingly applied in the medical field [4,5], demonstrating potential in patient education and clinical support roles [6-8]. Williams et al [9] showed that discharge summaries generated by GPT-4 were comparable in quality to those written by physicians. Gün [10] found high consistency between ChatGPT and emergency physicians in blood gas analysis interpretation. LLMs possess efficient text generation and information integration capabilities and can theoretically generate ward round record frameworks that comply with clinical standards, reducing physicians’ documentation workload [11]. However, for senior physician ward round records in emergency department wards—a document type requiring both timeliness and clinical complexity—validation based on real clinical data is lacking, particularly in Chinese-language medical documentation and the specialized field of acute poisoning [12].

DeepSeek, as a representative domestic LLM, features free use and strong Chinese-language comprehension capabilities [13]. This study, based on real clinical records from an emergency department, aimed to systematically compare the quality of initial senior physician ward round records generated by DeepSeek and ChatGPT against the quality of those written by human physicians using real acute poisoning cases across multiple evaluation dimensions. We hypothesized that LLMs would demonstrate comparable or superior performance to that of physicians in structured documentation tasks—particularly in information integration and differential diagnosis—while potentially showing deficiencies in complex clinical judgment domains such as definitive diagnosis formulation.

## Methods

### Study Design

This was a retrospective cross-sectional study. The LLM-generated records and blinded evaluations were conducted from September 2025 to December 2025 at the emergency department of Taihe Hospital, Hubei University of Medicine; the clinical records analyzed were from patients with acute poisoning admitted between January 2023 and December 2024.

### Ethical Considerations

The study protocol was approved by the hospital’s ethics committee (ethics approval number: 2025KS187). All medical record data were deidentified prior to analysis.

### Study Population

Inclusion criteria were (1) patients with acute poisoning admitted between January 2023 and December 2024; (2) complete medical records, including initial progress notes, attending physician ward round records, and initial senior physician ward round records; (3) hospital stay of 48 hours or longer; and (4) confirmed diagnosis of acute poisoning. Exclusion criteria were (1) incomplete medical records; (2) concurrent severe trauma, advanced cancer, or other critical conditions; and (3) chronic poisoning cases.

A total of 256 patient records were ultimately included and were categorized by poisoning type: 43% (n=110) cases of drug poisoning, 18.8% (n=48) cases of pesticide poisoning, 12.5% (n=32) cases of gas poisoning, 12.1% (n=31) cases of biological toxin poisoning, and 13.7% (n=35) cases of other poisoning (including food poisoning, alcohol poisoning, rodenticide poisoning, and chemical poisoning; Multimedia Appendix 1). The distribution of poisoning types reflects the actual clinical epidemiology of acute poisoning in our emergency department, with drug poisoning being the most prevalent category, consistent with national epidemiological trends [1]. No artificial balancing of the dataset was performed as the primary objective was to evaluate LLM performance under real-world, ecologically valid conditions.

### Ward Round Record Generation

**Figure 1.**
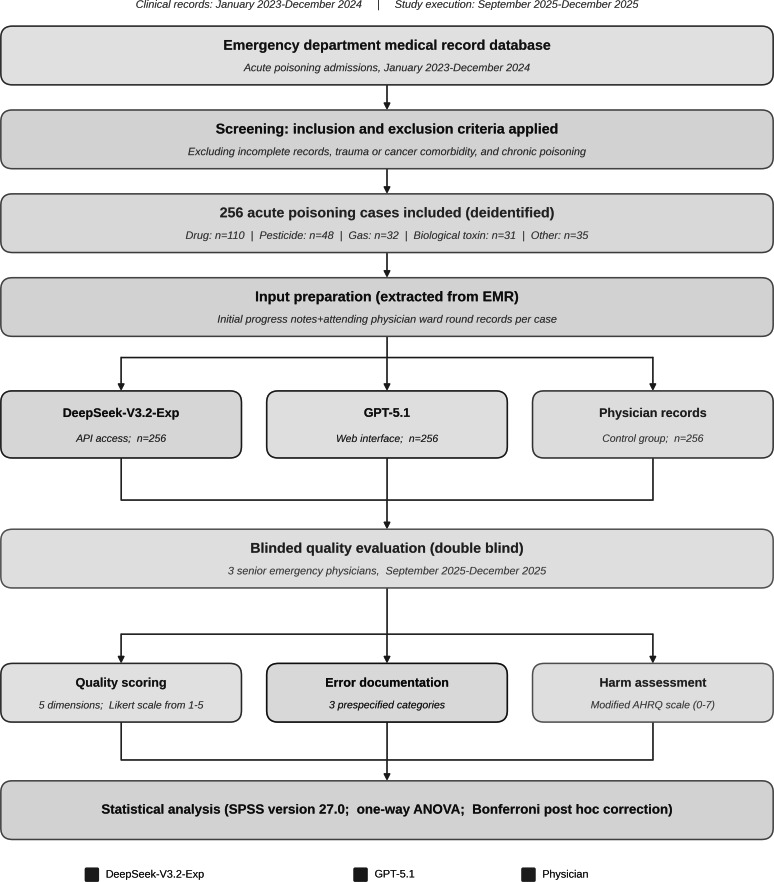
Research flowchart. AHRQ: Agency for Healthcare Research and Quality; API: application programming interface; EMR: electronic medical record.

The study workflow is shown in Figure 1. Initial progress notes and attending physician ward round records were extracted from the electronic medical record system as input data for each patient. DeepSeek-V3.2-Exp (November 2025 version) and GPT-5.1 (November 2025 version) were used to generate initial senior physician ward round records according to a standardized prompt template. All input data and prompts were in Chinese, consistent with standard clinical practice in our Chinese hospital setting, and all generated records were in Chinese, matching the language of the source medical records. The prompt design referenced the Basic Standards for Medical Record Writing issued by the National Health Commission of China on March 1, 2010. The original senior physician ward round records in the medical charts served as the control group.

DeepSeek-V3.2-Exp was accessed via an application programming interface (API), with parameters set to a temperature of 0.3, top_p of 0.95, max_tokens of 4096, frequency_penalty of 0, and presence_penalty of 0. GPT-5.1 was accessed through the official web interface with the memory function disabled, with conversation history cleared before each case to ensure session independence. The 2 LLMs were accessed via different methods owing to practical constraints during the study period: DeepSeek offered a stable API end point enabling systematic batch processing with controlled parameters, whereas API access to ChatGPT was not available to our research team, necessitating web interface use. A relatively low temperature value (0.3) was selected for DeepSeek to enhance output determinism and reduce the impact of randomness on documentation quality. All initial senior physician ward round records were generated during the final week of November 2025 to control for potential variation from model version updates. Each case was generated only once without repeated generation or manual selection to ensure objectivity of results.

### Evaluation Methods

The evaluation team consisted of 3 senior emergency physicians (attending physician level or above; mean experience 10 years). A double-blind design was used, with evaluators unaware of the record sources. Using the initial progress notes and attending physician ward round records as reference, evaluators scored both LLM-generated and actual senior physician ward round records on 5 dimensions: case characteristics, current diagnosis, differential diagnosis, treatment plan, and prognosis assessment. A self-designed senior physician ward round record scoring form was used as the evaluation tool (Table 1), with each dimension scored on a Likert scale from 1 to 5.

Error frequencies were documented under three prespecified categories: (1) inaccuracies—errors in how existing clinical information was processed or recorded, such as misattributed laboratory values, incorrect dates, or copying and pasting errors; (2) omissions—clinically relevant information present in the input data that was absent from the generated record; and (3) fabrications—content in the generated record that could not be traced to or was directly contradicted by the input clinical data, such as documenting a treatment not administered or a physical finding inconsistent with the patient’s actual examination. We use the term “fabrication” in preference to “hallucination” throughout this paper as it more precisely denotes the generation of clinically unsupported content. In physician-written records, fabrication-type errors corresponded to documentation of events or findings that did not occur. This taxonomy was adapted from established LLM error classification frameworks for clinical text [14,15]. Additionally, potential harm was assessed using a modified Agency for Healthcare Research and Quality harm scale [16].

**Table 1.** Evaluation dimensions of the senior physician ward round record scoring form^a^.

Dimension	Evaluation content
Case characteristics	Summarization of chief concern and present illness, integration of physical signs and auxiliary examinations, and summary of disease progression
Current diagnosis	Accuracy of primary diagnosis, completeness of diagnostic evidence, and rationality of diagnostic reasoning
Differential diagnosis	Comprehensiveness of differential diagnoses, sufficiency of differentiating evidence, and rationality of diagnostic exclusion
Treatment plan	Specificity of treatment regimen, plan for further investigations, and key points of condition monitoring
Prognosis assessment	Basis of prognostic judgment, identification of risk factors, and rationality of outcome prediction

a Each dimension was scored on a 5-point Likert scale: 1=“strongly disagree,” 2=“disagree,” 3=“neutral,” 4=“agree,” and 5=“strongly agree.”

### Statistical Analysis

Statistical analysis was performed using SPSS (version 27.0; IBM Corp). The intraclass correlation coefficient was used to assess interrater reliability, with mean scores used for subsequent analysis. Continuous variables were expressed as means and SDs with 1-way ANOVA for 3-group comparisons and Bonferroni correction for pairwise post hoc comparisons. Categorical variables were expressed as frequencies and percentages, with chi-square or Fisher exact tests for between-group comparisons. A *P* value of less than .05 was considered statistically significant.

## Results

### Interrater Reliability

Interrater reliability among the 3 evaluators was good, with an intraclass correlation coefficient for total scores of 0.839 (95% CI 0.819‐0.858; *P*<.001). Mean scores for each dimension and total scores were used for subsequent analysis.

### Total Score Comparison

**Figure 2.**
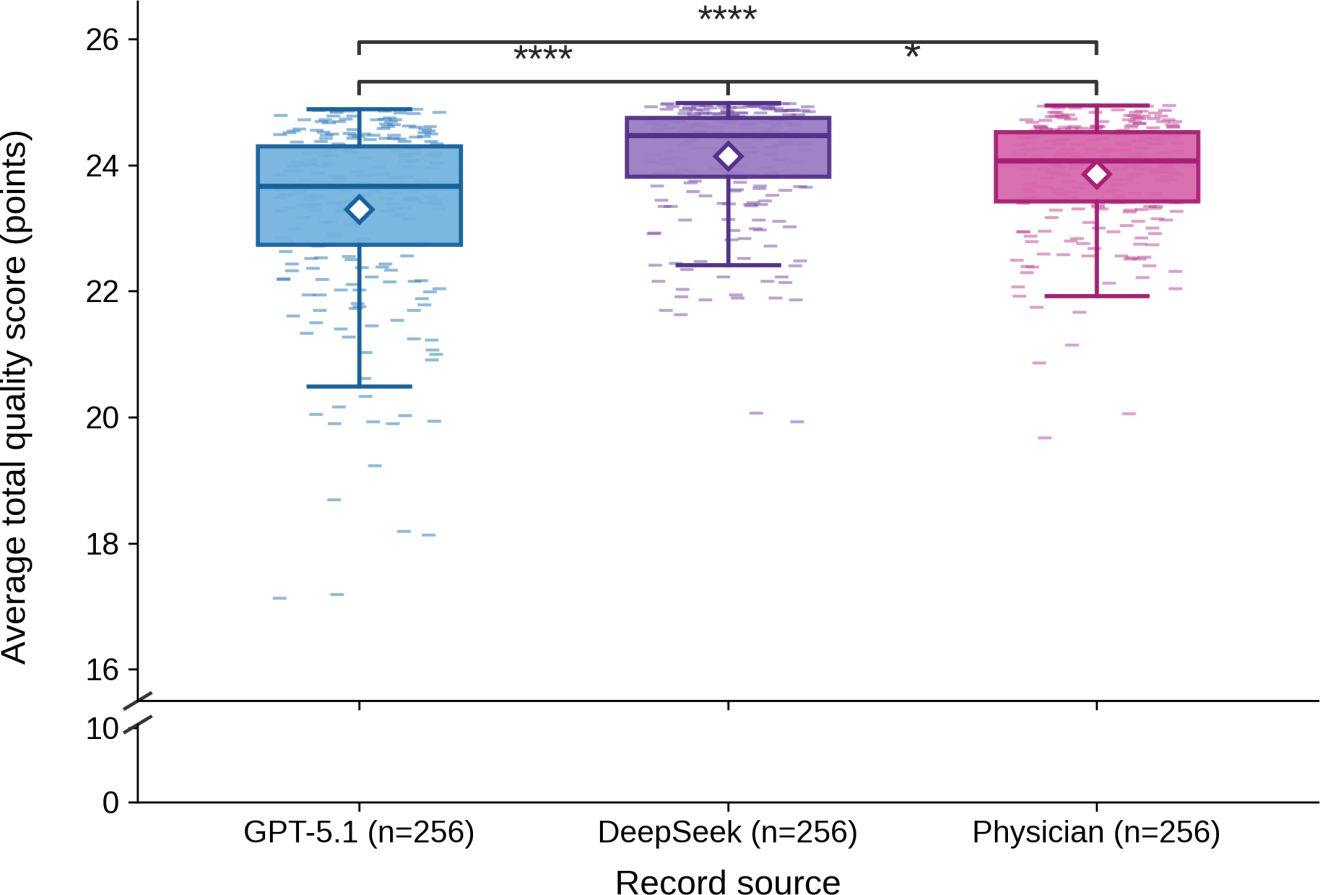
Comparison of total quality scores among GPT-5.1–, DeepSeek-V3.2-Exp–, and physician-written records. The boxes represent IQRs, the horizontal lines indicate medians, the diamonds denote means, and the whiskers extend to 1.5 times the IQR. Individual data points are shown as jittered dots. One-way ANOVA with Bonferroni post hoc correction was conducted. *P<.05; ****P<.0001.

Total scores differed significantly among the 3 groups (*F*_2,765_=38.918; *P*<.001). Bonferroni post hoc comparisons showed that the DeepSeek group achieved the highest total score, significantly higher than both the ChatGPT group and the physician group; the physician group’s total score was also significantly higher than that of the ChatGPT group (Figure 2 and Table 2).

**Table 2.** Comparison of scores across 5 dimensions among the 3 groups.

Dimension	GPT-5.1 score (n=256), mean (SD)	DeepSeek-V3.2-Exp score (n=256), mean (SD)	Physician score (n=256), mean (SD)	*F* test (*df*)^a^	*P* value
Case characteristics (range 1-5)	4.81 (0.41)	4.96 (0.23)^b^	4.90 (0.15)	18.27 (2, 765)	<.001
Current diagnosis (range 1-5)	4.43 (0.58)^c^	4.69 (0.43)^d^	4.86 (0.30)	57.94 (2, 765)	<.001
Differential diagnosis (range 1-5)	4.61 (0.73)^c^	4.98 (0.10)^d^	4.75 (0.36)	37.21 (2, 765)	<.001
Treatment plan (range 1-5)	4.81 (0.38)^c^	4.83 (0.37)	4.89 (0.30)	4.10 (2, 765)	.02
Prognosis assessment (range 1-5)	4.65 (0.47)^c^	4.73 (0.42)^c^	4.39 (0.54)	35.67 (2, 765)	<.001
Total score (range 5-25)	23.30 (1.42)^c^	24.14 (0.90)^d^	23.86 (0.86)	38.92 (2, 765)	<.001

a *F* statistic from one-way ANOVA.

b *P*<.05 vs the GPT-5.1 group (Bonferroni correction). By definition, this footnote does not appear in the “GPT-5.1” column.

c *P*<.05 vs the physician group. By definition, this footnote does not appear in the “Physician” column.

d Significance vs both groups.

### Comparison of Dimension Scores

As shown in Table 2, DeepSeek demonstrated optimal performance in both differential diagnosis (mean 4.98, SD 0.10) and prognosis assessment (mean 4.73, SD 0.42). DeepSeek’s case characteristics score (mean 4.96, SD 0.23) was statistically comparable to that of the physician group (mean 4.90, SD 0.15; *P*>.05), indicating no significant difference between them. The physician group achieved the highest scores in current diagnosis (mean 4.86, SD 0.30) and treatment planning (mean 4.89, SD 0.30). Notably, the physician group’s score in prognosis assessment (mean 4.39, SD 0.54) was significantly lower than that of both LLMs (*P*<.001).

### Subgroup Analysis by Poisoning Type

In drug poisoning cases, the DeepSeek group scored highest (mean 24.23, SD 0.75), with statistically significant differences in all pairwise comparisons. In pesticide poisoning cases, there was no significant difference between the DeepSeek and physician groups, but both scores were significantly higher than that of the ChatGPT group. In biological toxin poisoning cases, the physician and DeepSeek groups had similar scores, both significantly higher than those of ChatGPT. In gas poisoning and other poisoning cases, no statistically significant differences were observed among the 3 groups (Table 3).

**Table 3.** Subgroup analysis by poisoning type.

Poisoning type	Cases (n=256), n (%)	GPT-5.1 score (range 5-25), mean (SD)	DeepSeek-V3.2-Exp score (range 5-25), mean (SD)	Physicians score (range 5-25), mean (SD)	*F* test (*df*)	*P* value
Drug	110 (43)	23.34 (1.33)^a^	24.23 (0.75)^b^	23.79 (0.88)	21.39 (2, 327)	<.001
Pesticide	48 (18.8)	22.78 (1.33)^a^	23.92 (1.14)^c^	23.77 (1.00)	13.46 (2, 141)	<.001
Gas	32 (12.5)	24.29 (0.95)	24.42 (0.86)	23.95 (0.79)	2.50 (2, 93)	.09
Biological toxin	31 (12.1)	22.53 (1.86)^a^	23.97 (0.96)^c^	24.26 (0.62)	16.71 (2, 90)	<.001
Other	35 (13.7)	23.70 (1.03)	24.01 (0.88)	23.80 (0.73)	1.14 (2, 102)	.33

a *P*<.05 vs the physician group. By definition, this footnote does not appear in the “Physician” column.

b Significance vs both groups*.*

c *P*<.05 vs the GPT-5.1 group (Bonferroni correction). By definition, this footnote does not appear in the “GPT-5.1” column.

### Error Type Analysis and Potential Harm Assessment

Error frequencies and types across the 3 groups are shown in Tables4  5. Physician records had the lowest mean number of errors per record (1.38, SD 1.15), whereas DeepSeek (2.56, SD 1.72) and ChatGPT (2.31, SD 1.58) had significantly more errors (*P*<.001). Both LLMs showed significantly higher rates of omission errors (DeepSeek: mean 1.62, SD 1.25; ChatGPT: mean 1.45, SD 1.12) than the physician group (mean 0.68, SD 0.82; *P*<.001). Inaccuracy errors also differed significantly among groups (*P*=.008), whereas fabrication error rates showed no significant difference (*P*=.28).

**Table 4.** Analysis of error types across the 3 groups.

Error type	GPT-5.1, mean (SD)	DeepSeek-V3.2-Exp, mean (SD)	Physicians, mean (SD)	*F* test (*df*)	*P* value
Inaccuracies	0.62 (0.70)	0.68 (0.82)	0.42 (0.61)	4.85 (2, 765)	.008
Omissions	1.45 (1.12)^a^	1.62 (1.25)^a^	0.68 (0.82)	38.92 (2, 765)	<.001
Fabrications	0.24 (0.46)	0.26 (0.48)	0.28 (0.52)	1.28 (2, 765)	.28
Total errors	2.31 (1.58)^a^	2.56 (1.72)^a^	1.38 (1.15)	28.56 (2, 765)	<.001

a *P*<.001 vs the physician group (Bonferroni correction; see the Methods section for definitions of error categories).

**Table 5.** Examples of typical errors from the 3 groups.

Source and error type		Error description
DeepSeek-V3.2-Exp		
	Inaccuracy	The Poisoning Severity Score was systematically overestimated by 1 point relative to the actual severity in several cases.
	Omission	Omission of vital sign changes and positive findings from critical abdominal examinations.
	Fabrication	A superficial scratch on the arm of a patient with depression was attributed to a dermatological condition not present in the source record.
GPT-5.1		
	Inaccuracy	The correct diagnosis was included within the differential diagnosis list rather than as the primary diagnosis; the chief concern did not conform to standard medical record writing format.
	Omission	Omission of vital sign changes and positive findings from critical abdominal examinations.
	Fabrication	For a patient who was critically ill, first-level nursing care was ordered instead of the required special-level nursing care.
Physicians		
	Inaccuracy	Copying and pasting errors, such as transcribing “pleural effusion” as “pericardial effusion”; incorrect dosage units (mg vs g); and incorrect date entry.
	Omission	Failure to include certain minor findings from imaging studies in the diagnosis.
	Fabrication	Antihypertensive medications were not used during the actual treatment but were recorded as administered.

Overall, potential harm scores (measured on a scale from 0 to 7) were low across all 3 groups (Table 6; physician group: mean 0.45, SD 0.72; DeepSeek: mean 0.82, SD 0.95; ChatGPT: mean 0.71, SD 0.88; no statistically significant difference among groups: *F*=0.97 and *P*=.38). Harm scores per individual error also showed no significant difference (*P*=.56). However, the distribution of high-harm records (≥4 points) differed significantly (DeepSeek: 8/256, 3.1%; ChatGPT: 5/256, 2%; physician group: 2/256, 0.8%; *χ*^2^_2_=7.8; *P*=.02).

**Table 6.** Potential harm assessment results across the 3 groups^a^.

Indicator	GPT-5.1	DeepSeek-V3.2-Exp	Physicians	*P* value
Overall harm score, mean (SD)	0.71 (0.88)	0.82 (0.95)	0.45 (0.72)	.38
Harm score per error, mean (SD)	1.35 (1.01)	1.38 (1.05)	1.32 (0.98)	.56
High-harm records (≥4 points; n=256), n (%)	5 (2)	8 (3.1)	2 (0.8)	.02

a Harm scores were assessed using a modified Agency for Healthcare Research and Quality scale (0‐7 points): 0=no potential for harm; 1=potential for emotional distress or mild, transient discomfort; 2=potential for requiring additional treatment; 3=potential for temporary harm (bodily or psychological injury, likely not permanent); 4=potential for permanent harm; 5=potential for lifelong injury or disfigurement; 6=potential for severe permanent harm; and 7=potential for death*.*

## Discussion

### Summary of Findings

This study was the first to systematically compare initial senior physician ward round records written by DeepSeek, ChatGPT, and physicians for patients with acute poisoning using real clinical progress notes and attending physician records as structured input. By grounding the evaluation in authentic clinical data rather than simulated scenarios, this design may offer greater ecological validity than studies using hypothetical vignettes, although the single-center retrospective nature limits broader generalizability.

Overall, DeepSeek achieved the highest total score, significantly outperforming both ChatGPT and the physician group. This finding demonstrates that LLMs have acquired a basic capability for assisting in medical documentation generation and that the domestic model DeepSeek performs comparably or superiorly to the mainstream international model ChatGPT—providing empirical support for its application in Chinese clinical settings. With advantages including free access, absence of data export requirements, and strong alignment with Chinese medical documentation conventions, DeepSeek has broad potential in domestic medical documentation assistance [13].

### Dimension-Level Performance

The LLMs—particularly DeepSeek—demonstrated outstanding performance in case characteristic summarization and differential diagnosis, reflecting their powerful information extraction and structured knowledge retrieval capabilities [17,18]. In the differential diagnosis dimension, DeepSeek achieved near-perfect scores (mean 4.98, SD 0.10), comprehensively listing diseases requiring differentiation and providing sufficient differentiating evidence. However, in current diagnosis—a domain requiring integrative clinical judgment—the physician group maintained a significant advantage (mean 4.86, SD 0.30). Accurate clinical diagnosis depends not only on standardized medical knowledge but also on the synthesis of individual patient circumstances, recognition of atypical presentations, dynamic tracking of disease evolution, and the weighing of complex comorbidities. This capacity for contextual clinical reasoning, built through accumulated experience, represents a core domain in which current LLMs cannot fully substitute trained clinicians [19]. Notably, GPT-5.1 demonstrated a pattern of placing the correct diagnosis within the differential diagnosis list rather than as the primary diagnosis [20]—a finding consistent with results reported for GPT-4 by McDuff et al [21].

Both LLMs scored significantly higher than the physician group in prognosis assessment. This may reflect the models’ tendency to generate more comprehensive assessment frameworks encompassing risk factors and outcome scenarios [22], or it may be attributable to clinicians simplifying prognosis documentation under time pressure in routine practice. Regardless, the clinical accuracy of LLM-generated prognostic content requires prospective validation against actual patient outcomes before it can be relied upon clinically.

### Subgroup Performance by Poisoning Type

Subgroup analysis revealed heterogeneity in LLM performance, with advantages concentrated in categories characterized by standardized information and well-established management protocols. In drug poisoning—the most common category—DeepSeek performed most prominently, consistent with the hypothesis that structured, protocol-amenable scenarios favor LLM pattern matching and knowledge extraction.

In pesticide and biological toxin poisoning cases, ChatGPT scored significantly lower than both DeepSeek and the physician group, whereas DeepSeek and physicians performed comparably. Pesticide and biological toxin poisonings (such as wasp stings) are more prevalent in China and involve complex toxicological mechanisms, diverse clinical presentations, and individualized treatment strategies. DeepSeek’s maintained performance in these categories may reflect its deeper integration with domestic toxicology databases, local clinical guidelines, and specialized knowledge during training.

In gas poisoning (primarily carbon monoxide poisoning), no significant differences were observed among groups, possibly reflecting the highly standardized treatment principles for this category (eg, hyperbaric oxygen therapy) and the relatively small subgroup sample size. In the heterogeneous “other poisoning” category, the absence of significant differences suggests that current LLMs may have limitations in handling complex, atypical cases. These findings reinforce the recommendation that LLMs function as documentation assistants, with all generated records subject to systematic review by senior physicians before clinical use.

### Comparison With Existing Literature

The findings of this study are broadly consistent with those of Schwieger et al [23], confirming that LLMs possess significant advantages in medical documentation standardization and efficiency. Unlike prior studies focused on radiology reports and discharge summaries [24-26], this study specifically addressed emergency department ward round records, extending the evidence on LLM applicability to critical care documentation. Consistent with Hains et al [27], who identified performance gaps when processing real electronic medical record data, our study found suboptimal LLM performance in complex and atypical poisoning categories. The finding that fine-tuned models may outperform general models in specific diagnostic tasks [18] further suggests that domain-specific adaptation could improve clinical performance in specialized fields such as toxicology.

Regarding safety, existing evidence indicates that LLM-drafted discharge summaries have comparable overall quality and safety to those of physician-written records [9]; however, high-harm record rates in this study were higher for LLMs than for physicians, suggesting that ward round records—which encode more complex clinical reasoning than discharge summaries—may require additional scrutiny. This is consistent with broader evidence that LLM limitations are more pronounced in complex clinical decision-making contexts [28-30].

### Safety Considerations

Although overall potential harm scores were low across all 3 groups, the significantly higher proportion of high-harm records (≥4 points) in the LLM groups—3.1% (8/256) for DeepSeek and 2% (5/256) for ChatGPT vs 0.8% (2/256) for the physician group (*χ*^2^=7.8; *P*=.02)—carries important clinical safety implications. High-harm errors included systematic PSS score overestimation leading to overtreatment risk, inappropriate downgrading of nursing care levels for patients who were critically ill, and omission of critical physical examination findings. These may reflect LLM overconfidence in atypical cases, provision of definitive recommendations without acknowledging diagnostic uncertainty, or fabrication errors at critical decision points such as antidote dosing or nursing level assignment.

These findings underscore the irreplaceable role of physician review in human–artificial intelligence collaboration models. We recommend the following safety mechanisms for clinical implementation: (1) mandatory senior physician review of all LLM-generated ward round records prior to incorporation into official documentation, (2) enhanced review protocols for critically ill patients and complex poisoning types, and (3) establishment of ongoing error monitoring and feedback systems to enable continuous optimization of model application. Notably, although physician-written records had the lowest proportion of high-harm errors, their occurrence (2/256, 0.8%) confirms that documentation quality control is necessary regardless of record authorship, primarily to address copy-paste errors and dosage unit confusion.

### Limitations

Several limitations warrant consideration. First, this was a single-center retrospective study; the findings may not generalize to institutions with different patient populations, clinical workflows, or regional epidemiological profiles. Second, the retrospective design precluded evaluation of actual documentation time or work efficiency improvements, which are clinically important considerations given the timeliness demands of emergency ward round documentation. Third, patient outcome data were not linked, so the clinical impact of documentation errors could not be directly assessed. Fourth, sample sizes in some subgroups were relatively small, potentially limiting statistical power. Fifth, the 2 LLMs were accessed via different methods—API for DeepSeek and web interface for ChatGPT owing to API unavailability—which may have introduced minor differences in output consistency. Sixth, the conservative temperature setting (0.3) for DeepSeek may have constrained output diversity; the effects of alternative parameter configurations were not explored. Seventh, the number of high-harm records was small, limiting in-depth analysis of associated risk factors. Future studies should address these limitations through multicenter prospective designs, time efficiency measurements, outcome linkage, and investigation of different model parameter settings. Additionally, the inference that language differences minimally impact LLM output [31] was drawn from medical examination studies using an earlier ChatGPT version, and its applicability to real-world clinical notes with the current model versions should be interpreted cautiously.

### Conclusions

LLMs demonstrated satisfactory performance in generating initial senior physician ward round records in the emergency department, with DeepSeek particularly outperforming the physician group in case characteristic integration and differential diagnosis. However, physician-level performance was not achieved in core clinical diagnosis, and the significantly higher proportion of high-harm records in LLM-generated documentation underscores the necessity of rigorous physician review mechanisms in any clinical implementation. These findings position LLMs as documentation assistants rather than independent clinical authors, with the potential to augment physician efficiency without supplanting clinical judgment. Realizing this potential safely will require standardized quality control frameworks, including mandatory senior physician review, heightened oversight for complex or critically ill cases, and continuous monitoring of high-harm errors. Future research should prioritize multicenter prospective studies, domain-specific model fine-tuning for clinical toxicology, integration of time efficiency metrics, and the development of validated quality standards for LLM-generated medical records in emergency settings.

## Supplementary material

10.2196/91222 Multimedia Appendix 1 Clinical characteristics of the 256 included acute poisoning patients.

## Abbreviations

API: application programming interface
LLM: large language model
