# Supplementary material for: Quality Evaluation of Large Language Model–Assisted Generation of Initial Senior Physician Ward Round Records for Patients With Acute Poisoning: Cross-Sectional Study
Source: J Med Internet Res. 2026 Jun 30;28:e91222. doi: 10.2196/91222 (PMC13318081; doi:10.2196/91222)
Supplement: Multimedia Appendix 1 [file jmir-v28-e91222-s001.docx]

**Supplementary Table 1.** Clinical characteristics of 256 poisoning patients

| **Variable** | **Number of Cases**  **(n)** | **Proportion**  **(%)** | **Mortality Rate**  **(%)** |
| --- | --- | --- | --- |
| Type of Poisoning |  |  |  |
| Drug Poisoning | 110 | 42.97 | 8.6 |
| Pesticide Poisoning | 48 | 18.75 | 48.6 |
| Gas Poisoning | 32 | 12.50 | 2.1 |
| Biological Toxin Poisoningt | 31 | 12.11 | 5.3 |
| Other Poisoning^a^ | 35 | 13.67 | 5.6 |
| **Gender** |  |  |  |
| Male | 142 | 55.47 | - |
| Female | 114 | 44.53 | - |
| **Age (years, mean ± SD)** | 45.3 ± 18.7 | - | - |
| **Length of Hospital Stay (days, mean ± SD)** | 6.8 ± 4.2 | - | - |
| **PSS Score^b^** |  |  |  |
| Mild (0–1 point) | 89 | 34.77 | 0 |
| Moderate (2 points) | 102 | 39.84 | 2.9 |
| Severe (3 points) | 65 | 25.39 | 27.7 |

*Note: a, Other poisonings include food poisoning, alcohol poisoning, rodenticide poisoning, detergents, and metal poisoning, among others; b,PSS = Poisoning Severity Score;*
